# Polyphenols profile and antioxidant activity of skin and pulp of a rare apple from Marche region (Italy)

**DOI:** 10.1186/1752-153X-8-45

**Published:** 2014-07-10

**Authors:** Giovanna Giomaro, Anastasia Karioti, Anna Rita Bilia, Anahi Bucchini, Laura Giamperi, Donata Ricci, Daniele Fraternale

**Affiliations:** 1 Dipartimento di Scienze della Terra, della Vita e dell’Ambiente, Università degli Studi di Urbino "Carlo Bo", Via Bramante 28, 61029 Urbino, PU, Italy; 2 Dipartimento di Chimica Edificio di Scienze Farmaceutiche, Università di Firenze, Via U. Schiff 6, 50019 Sesto Fiorentino, FI, Italy; 3 Dipartimento di Scienze Biomolecolari, Università degli Studi di Urbino "Carlo Bo", Via Bramante 28, 61029 Urbino, PU, Italy

**Keywords:** Pelingo apple, Antioxidant activity, Red flesh apple, HPLC-DAD-MS, ORAC, Cyanidin-3-O-galactoside, Human health

## Abstract

**Background:**

Apples are an important source of polyphenols in the human diet and the consumption of this fruit has been linked to the prevention of degenerative diseases.

**Results:**

Catechins, procyanidins, hydroxycinnamic acids, flavonol glycosides, dihydrochalcone glycosides and one anthocyanin: cyanidin-3-O-galactoside, were identified both in the peel and pulp. Procyanidins, catechins and flavonols represent the main constituents of peel. Concerning the antioxidant activity, in the reduction of the stable DPPH radical and in the inhibition of lipid peroxidation, the ethanolic extracts of red peel and red pulp showed a good similar activity comparable to ascorbic acid in the DPPH test and about ten times more active than BHT in the lipoxygenase test, and were much more active than aqueous extracts. The ORAC value of red pulp aqueous extract resulted comparable to that of red berries: vaccinium, rubus and ribes, foods appreciated for their health value.

**Conclusion:**

This apple contains an appreciable amount of polyphenols also in the flesh; this variety with red flesh can also be useful for researchers engaged in apples varietal innovation in addition to being used as food apple.

## Background

The genus *Malus* is native to the temperate zones of the northern hemisphere, Europe, Asia, and North America, and is comprised of about 30–35 species of small deciduous trees or shrubs in the Rosaceae family. The domesticated, table apple, *Malus x domestica* Barkh. is considered to be a complex interspecific hybrid. The main ancestor is thought to be *Malus sieversii* M. Roem [1,2] along with other ancestors, those being *Malus sylvestris* Mill., *Malus pumila* Mill. and *Malus dasyphylla* Borkh [3]. The ancestors are generally known as "wild apples", name derived from their typically small and tart fruits [4,5]. Among the ancestors, *Malus pumila* Mill. produces fruits that show red coloration in both the skin and flesh. It is tart, relatively nonjuicy, with a small size and oxidizes easily and therefore not used in human food [6]. We know that fruits and vegetables contain many compounds including phenolics, thiols, carotenoids, tocopherols and glucosinolates, which may protect against cardiovascular diseases, cancer and cataracts [7]. This protective property of vegetables and fruits is thought to depend on their contents of bioactive antioxidant compounds that exert a scavenging activity towards free radicals which are thought to be responsible for many age related diseases [8]. Apples are an important source of polyphenolics which are responsible for most of the antioxidant activities of the fruit, far over the amount explained by the presence of ascorbic acid [9]. It was confirmed that a regular use of apples in a diet contributes in a significant way to the intake of polyphenols [10,11]. The consumption of apples has been linked to the prevention of degenerative diseases; a reduction in the risk of lung cancer, asthma, type-2 diabetes, thrombotic stroke, ischemic heart disease, and antiproliferative activities have been attributed to apple consumption [12,13]. In this work, for the first time, we studied the content of total polyphenols, total anthocyanins, and the *in vitro* antioxidant activity of the extract of a rare red Italian wild apple named apple "Pelingo". The fruit of this species is similar to that of *Malus pumila* Mill. having red skin and flesh but covered in bloom, juicy, fragrant, not tart, with size comparable to the known table apples [14]. Currently, apples with red flesh and sweet-fruity flavor have not yet appeared on the market. Researches on the production of new varieties of apples (no-OGM) (Organisms Genetically Modified) with such properties are still being performed by academic institutions, agricultural companies or consortia which through varietal selections, breeding and pollination of different varieties of apples, attempt to obtain a market production.

The distinctive characteristic of "Pelingo", having unknown origin is related to the red colour of pulp and the fruity- sweet flavour [14,15].

## Results and discussion

### HPLC-DAD-MS analysis

In the present study ethanol and aqueous extracts of peel and pulp of "Pelingo" apples were prepared and evaluated for their chemical profile using HPLC-DAD-MS analysis. Different HPLC columns available in our lab were tested in order to find the most appropriate for this kind of extracts: a Luna® (150 × 4.6 mm, 5 μm; Phenomenex; flow 1.0 ml/min), Zorbax Eclipse plus® (150 × 3 mm, 3.5 μm; Agilent; flow 0.4 mL/min) and a Zorbax SB-Aq® (150 × 3 mm, 3.5 μm; Agilent; flow 0.3 mL/min). Gradient conditions were different in each case, adjusted to the dimensions and demands of each stationary phase (mixture of acetonitrile and water acidified by HCOOH at pH 3.2). Among the columns tested the Zorbax SB-Aq® gave the best results concerning the separation of the procyanidins. However, under these experimental conditions (pH = 3.2) detection of anthocyanins is not favoured, as this type of constituents requires higher pH values. A Synergi max RP®, Phenomenex allowed the use of highly acidic solvents (5% HCOOH, pH = 2) necessary to stabilize the anthocyanins and increase the anthocyanin absorption bands at 520 nm [16], while it provided a satisfactory separation of the rest of the constituents.

**Figure 1:**
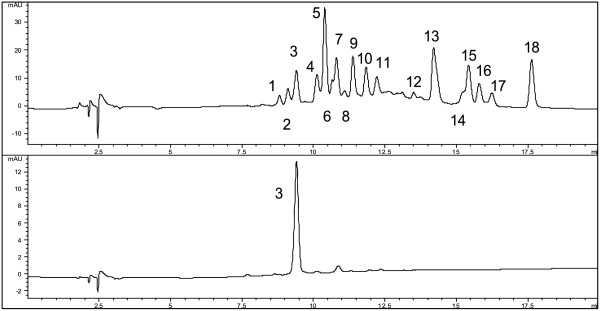
HPLC/DAD chromatogram of the ethanol extract at 280 and 520 nm.

The constituents of the extracts were identified mainly by UV and MS spectral data. The chromatographic profiles of each extract were similar even if anthocyanins were present only in traces in the aqueous extracts. For this reason the ethanol extract of the peel was used as the most representative sample for the identification of the peaks in the chromatograms. In Figure 1 is reported the HPLC/DAD chromatogram of the ethanol extract at 280 and 520 nm. Data concerning identification of the peaks are shown in Table 1, where the retention time, UV–vis absorptions and electrospray ionization mass spectrometry in both positive and negative ion mode of all the compounds are reported. Positive ionization mode at 120 eV gave the best results for the identification of anthocyanins, whereas negative ionization was more suitable for the rest of the phenolic constituents.

**Table 1:** UV and MS data obtained under negative and positive ionisation mode of the extracts

	**Rt (min)**	**UV (nm)**	**MS**	**Identification**	**Mode of identification**
**1**	8.8	278	289, **577** [M-H]^-^/**579** [M + H]^+^	procyanidin (dimer)	MS/UV + ref
**2**	9.1	280	289, 577, **865** [M-H]^-^/**867** [M + H]^+^	procyanidin (trimer)	MS/UV + ref
**3**	9.5	278, 518	**447** [M-H]^-^/**449** [M + H]^+^	cyanidin-3-O-galactoside	std + MS/UV
**4**	10.1	280	**289** [M-H]^-^/**291** [M + H]^+^	catechin	std + MS/UV
**5**	10.4	292, 328	191, **353** [M-H]^-^/**355** [M + H]^+^	chlorogenic acid	std + MS/UV
**6**	10.7	270, 304sh	**289** [M-H]^-^/**291** [M + H]^+^	epicatechin	std + MS/UV
**7**	10.8	278	577, **865** [M-H]^-^/**867** [M + H]^+^	procyanidin (trimer)	MS/UV + ref
**8**	11.1	278	577, **865** [M-H]^-^/579, **867** [M + H]^+^	procyanidin (trimer)	MS/UV + ref
**9**	11.4	278	577, 865, **1153** [M-H]^-^/579, 867, **1155** [M + H]^+^	procyanidin (tetramer)	MS/UV + ref
**10**	11.8	278, 312sh	191, **337** [M-H]^-^/**339** [M + H]^+^	p-coumaroylquinic acid	
**11**	12.2	278, 312sh	**337** [M-H]^-^/**339** [M + H]^+^	p-coumaroylquinic acid	MS/UV + ref
**12**	13.5		**609** [M-H]^-^/303, **611** [M + H]^+^	rutin	std + MS/UV
**13**	14.2	254, 264, 354	**463** [M-H]^-^/303, **465** [M + H]^+^	hyperoside + quercetin-3-O-glycoside	std + MS/UV
**14**	15.2	258, 356	**433** [M-H]^-^/303, **435** [M + H]^+^	quercetin-3-O-pentoside	MS/UV + ref
**15**	15.4	284	433, **567** [M-H]^-^/275, 435, **569** [M + H]^+^	phloretin-2-xyloglycoside	MS/UV + ref, tentatively
**16**	15.8	266, 356	**433** [M-H]^-^/303, **435** [M + H]^+^	quercetin-3-O-pentoside	MS/UV + ref
**17**	16.2	266, 356	**447** [M-H]^-^/303, **449** [M + H]^+^	quercitrin [quercetin-3-O-rhamnoside]	std + MS/UV
**18**	17.6	284	**435** [M-H]^-^/275, **459** [M + Na]^+^	phloretin-2-glycoside	MS/UV + ref, tentatively

The pseudomolecular ions are indicated in boldface.

In the chromatogram seven peaks of catechins and procyanidins (**1**, **2**, **4**, **6**, **7**, **8** and **9**) were present, having common UV characteristics with a maximum absorbance at ~ 280 nm. The MS analysis of these peaks revealed fragmentation patterns typical of catechins and procyanidins. In the latter case the progressive loss of the monomeric units of catechin (of weight ca 289 dalton) was observed: for the dimer at 8.8 min a second fragment at *m/z* = 289 [M-289]^-^ (negative ionization mode) was observed, whereas for trimers, apart their pseudomolecular peaks at *m/z* = 865 [M-H]^-^, fragments at *m/z* = 577 [M-289]^-^ were evidenced (peaks **1**, **2**, **7** and **8**). Peak **5** had quasi-molecular fragments at 353 [M-H]^-^ and 355 [M + H]^+^*m/z* and the characteristic ion at *m/z* 191 [M-162-H]^-^, (Table 1). This ion is typical of the presence of the quinic acid moiety. Peak **5** was attributed to chlorogenic acid. Its UV profile was typical of a caffeoyl derivative with maxima at 292 and 328 nm. Its presence was further confirmed by use of reference standard and was the predominant phenolic acid. At higher retention times (11.4 and 11.8 min), in agreement with the elution order two minor peaks (**10**, **11**) were assigned to the less polar coumaroylquinic acids. Their presence in apple peels has been previously confirmed in other apple varieties [17,18]. Both of them gave a characteristic band at 312 nm indicating the change of the type of the phenolic acid attached and common quasi-molecular ions at *m/z* = 337 [M-H]^-^ and 191 [M-147-H]^-^, suggesting the presence of one coumaroyl unit (instead of caffeoyl) and a quinic acid.

Actually five flavonoid glycosides were detected and identified in the extracts (Figure 1), belonging to flavonol derivatives. Their UV spectra exhibited two major absorption peaks in the regions of 355 nm (Band I) and 255–266 nm (Band II). Their MS spectral data gave evidence of the presence of quercetin (*m/z* = 303, [M-sugars + H]^+^) as the aglycone in all cases.

In the identification of dihydrochalcone glycosides, two dihydrochalcones were detected, and tentatively identified by comparison with literature data as phloretin-2′-*O*-xyloglucoside and phloretin-2′-*O*-glucoside (phloridzin) at 15.4 and 17.6 min, respectively. Their UV spectra were very similar to those of procyanidins with only one maximum at 284 nm, a wavelength slightly lower than that of procyanidins which permits their discrimination. The mass spectra of phloretin-2′-*O*-xyloglucoside presented pseudomolecular ions at *m*/*z* = 569 [M + H]^+^ (positive ionization mode) and 567 [M-H]^-^ (negative ionization mode) and fragments at *m/z* = 435 [M-xylosyl]^+^ and 275 [A + H]^+^ (phloretin aglycone) indicating the successive loss of the xylose and glucose units, respectively. Mass spectra of phloretin-2′-*O*-glucoside exhibited similar peaks.

Finally, as to the identification of anthocyanins, under the strong acidic conditions (pH = 2) during the present analysis anthocyanins exist primarily in the red coloured form of flavylium cations and give a strong absorption maximum at 520–535 nm (peak **3**, Figure 1). In the studied samples cyanidin-3-*O*-galactoside was identified as the only anthocyanin. Its mass spectra in the positive ion mode exhibited a molecular ion [*M*] + at *m*/*z* 449. Its identification was confirmed by use of reference standard.

**Figure 2:**
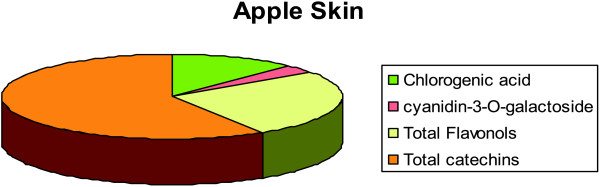
Constituents of skin.

For the quantification of the constituents by HPLC-DAD, only the richest extracts (ethanol extracts of skin and pulp) were taken in consideration. Aqueous extracts had a very low content in phenols and under the experimental conditions, the concentration was close to the LOQ and LOD limits of the analysis, due to the lower yield. Quantification was carried out expressing procyanidins as catechin, flavonols as quercetin-3-O-glycoside, whereas chlorogenic acid and cyanidin-3-O-galactoside, as such. In Table 2 the results of the quantitative analyses are reported. It is clearly observed that skin extracts are more abundant in phenolic constituents, in contrast to apple pulp. Main constituents in the apple pulp are chlorogenic acid and cyanidin-3-O-galactoside, the rest of the constituents were in traces (not quantifiable) or not observed. Results are better depicted in Figure 2.

**Table 2:** Amounts of constituents in the studied ethanol extracts

**Constituent**	**Skin extract mg/100 mg (RSD)**	**Pulp extract mg/100 mg (RSD)**	**Skin mg/100 g (RSD)**	**Pulp mg/100 g (RSD)**
Chlorogenic acid	1.53 ± 0.01 (0.33)	0.37 ± 0.01 (0.25)	49.82 ± 0.17 (0.33)	13.63 ± 0.04 (0.29)
cyanidin-3-O-galactoside	0.41 ± 0.03 (0.67)	0.017 ± 0.001 (0.94)	13.37 ± 0.09 (0.67)	0.63 ± 0.01 (0.94)
rutin	0.13 ± 0.01 (1.16)	-	4.07 ± 0.06 (1.55)	-
hyperoside + quercetin-3-O-glycoside	1.85 ± 0.01 (0.36)	-	59.9 ± 0.13 (0.21)	-
quercetin-3-O-pentoside	0.34 ± 0.03 (0.49)	-	10.96 ± 0.04 (0.34)	-
quercetin-3-O-pentoside	0.69 ± 0.01 (0.75)	-	22.56 ± 0.24 (1.06)	-
quercitrin [quercetin-3-O-rhamnoside]	0.38 ± 0.01 (0.38)	-	12.33 ± 0.05 (0.41)	-
Total Flavonols (RSD)	3.38 ± 0.01 (0.38)	-	109.83 ± 0.41 (0.38)	-
Dimer (**1**)	0.42 ± 0.01 (1.07)	-	13.76 ± 0.15 (1.07)	-
Trimer (**2**)	0.71 ± 0.02 (3.61)	-	23.15 ± 0.83 (3.61)	-
Catechin (**4**)	0.96 ± 0.02 (1.74)	-	31.34 ± 0.54 (1.74)	-
Epicatechin (**6**)	1.97 ± 0.06 (3.19)	-	63.42 ± 1.79 (2.82)	-
Trimer (**7**)	0.81 ± 0.02 (2.33)	-	26.30 ± 0.61 (2.33)	-
Trimer (**8**)	1.50 ± 0.03 (1.89)	-	48.62 ± 0.92 (1.89)	-
Tetramer (**9**)	1.25 ± 0.01 (0.46)	-	40.63 ± 0.19 (0.46)	-
Total catechins (RSD)	7.61 ± 0.08 (1.03)	-	247.21 ± 2.55 (1.03)	-
TOTAL POLYPHENOLS			420.2	

The developed HPLC analytical system in our work provided good resolution of the constituent profile and led to the identification of the majority of the constituents. Aqueous and ethanolic extracts of peel and pulp were tested by HPLC-DAD-MS, showing similar qualitative fingerprints and being the ethanol extract of peel the richest one. Overall, 18 compounds were identified and quantified by their UV and MS data and in many cases by comparison their Rf with a reference standard. The identified constituents, belonging to four representing classes of polyphenols: quinic acid derivatives, procyanidins, flavonols and anthocyanins, have been already identified in many apple varieties [8]. Novelty is mainly represented by the presence of appreciable quantities of cyanidin-3-O-galactoside not only in the peel, but also in the pulp, as resulting from the red colour.

### Total anthocyanin and polyphenols content

The total anthocyanin content of the apple extracts was measured using the differential pH method reported by Elisia et al. [19] and Tzulker et al. [20], while polyphenols were evaluated using the Prussian Blue method [21].

The peel and flesh of our red apple contains 124.60 ± 9.23 μg/g FW and 53.94 ± 8.64 μg/g FW of total anthocyanins after the extraction in 80% ethanol, and 11.53 ± 1.36 μg/g FW and 6.29 ± 0.53 μg/g FW after the extraction in H_2_O (Table 3).

**Table 3:** Total polyphenols, anthocyanins and antioxidant activity of tested extracts

	**Total polyphenols mg/g FW**	**Total anthocyanins μg/g FW**	**DPPH assay EC**_**50 **_**mg DW/mL**	**Lipoxygenase assay IC**_**50 **_**μg DW/mL**	**ORAC μmol Trolox eq./g DW**
Ethanolic extract (peel)	3.21 ± 0.26^c^	124.60 ± 9.23^c^	0.10 ± 0.02^a^	0.10 ± 0.01^a^	44.07 ± 3.74^c^
Ethanolic extract (flesh)	1.67 ± 0.15^b^	53.94 ± 8.64^b^	0.13 ± 0.01^a^	0.33 ± 0.02^b^	23.19 ± 2.41^a^
Aqueous extract (peel)	1.45 ± 0.17^b^	11.53 ± 1.36^a^	0.59 ± 0.05^b^	446 ± 48.7	31.99 ± 3.52^b^
Aqueous extract (flesh)	0.36 ± 0.04^a^	6.29 ± 0.53^a^	0.58 ± 0.03^b^	3688 ± 35.27	42.97 ± 4.73^c^
BHT			0.087 ± 0.012	3.86 ± 0.25	
Trolox			0.007 ± 0.001	11.89 ± 1.22	
Ascorbic acid			0.110 ± 0.007	18.63 ± 1.31	

The values are the average of three determinations (+ - SD). Significant differences among the means are evaluated using Bonferroni's multiple comparison test. Values with the same letters in a column are not significantly different at the 0.05 probability level. The letters are given following increasing values.

Table 3 shows the results obtained and analyzed with statistical analysis (ANOVA).

Comparing the anthocyanins content in peel and flesh aqueous extracts not significant statistical differences were revealed. However, values statistically different in ethanolic extracts from the flesh and peel were recorded. Significant differences are always present when comparing two different types of extracts obtained from the same part of the fruit.

The peel and flesh of our red apple contains 3.21 ± 0.26 mg/g FW and 1.67 ± 0.15 mg/g FW of total polyphenols after the extraction in 80% ethanol, and 1.45 ± 0.17 mg/g FW and 0.36 ± 0.04 mg/g FW after the extraction in H_2_O (Table 3).

Concerning the content of total polyphenols a statistical difference between the different parts of the fruit in each of the two types of extracts was detected. In fact, the values obtained for the peel and flesh ethanolic extract showed statistical differences. The same result was obtained for aqueous extracts.

No statistical difference was observed when comparing only the peel aqueous extract and the flesh ethanolic extract,showing that the highest content of polyphenols is present in the skin.

Regarding the total anthocyanins content extracted with 80% ethanol, our apples contain in the peel more total anthocyanins than Ida Red cultivar and the anthocyanins content in the flesh of our red apples is major than the total anthocyanins detected in the peel of red apples best known in the market.

Curiously, the apple we studied showed an interesting content of anthocyanins also in the flesh that is red and this, in our opinion, is very important from a nutritional point of view, considering the anthocyanins antioxidant activity.

It is known that the health-protection properties of apples have been attributed to the presence of polyphenols, rather than to ascorbic acid [22]. Although ascorbic acid has been found to be the most abundant vitamin in apples, its occurrence is about 10–200 times lower than that usually reported for polyphenols [23].

### DPPH assay

Leontowicz et al. [24] report that the ethanolic extract of *Malus domestica* Borkh var. Golden Delicious showed the greatest activity to quench DPPH radicals; the apple peel extract at the level of 5 mg/mL quenched 98% of DPPH radicals, in contrast the apple pulp extract at the same concentration quenched only 54.4% of DPPH radicals.

A very good correlation was observed between the antioxidant potentials determined by DPPH and the total polyphenols in agreement with Chinnici et al. [8] who used the DPPH test to determine the radical scavenging activities of peels and pulps of Golden Delicious apples cv. According to Chinnici et al. [8] data, the total antioxidant capacity values of peels were about 2.5 times higher than those found in pulps, probably due to the higher content of polyphenols in the skin.

The use of DPPH was also reported by Hamauzu et al. [25] in the examination of a Japanese apple cultivar (Fuji) and by Lamperi et al. [26] in four Italian popular apple varieties: Golden Delicious, Annurca, Red Chief and Staynam Neepling.

In line with the higher abundance of polyphenols and total anthocyanins in peel, the authors show in all cases higher radical scavenging activity values for the peel extracts with the exception of the Golden Delicious cultivar, while the values of radical scavenging activity for the flesh extract (all are white-fleshed cultivars) do not show significant differences between them and are lower than the values reported for the peel extracts.

Finally, even Iacopini et al. [27] who evaluated the biological properties of old Italian apple cultivars, support that the antioxidant activity of various parts of apples was positively correlated with total polyphenolic concentration and with the concentration of the principal phenolic compounds present in apple extracts such as the anthocyanins always more present in the peel than in the pulp.

In the reduction of the stable radical DPPH the activity (expressed as EC_50_) of the ethanolic extract of peel, 0.10 ± 0.02 mgDW/mL, and pulp 0.13 ± 0.01 mgDW/mL of our red apple was comparable to ascorbic acid 0.11 ± 0.0073 mgDW/mL (Table 3).

It is noteworthy that in the present study no differences between the radical scavenging activity of peel and pulp were found. The pulp of the investigated red apple contains a high concentration of anthocyanins, 53.94 ± 8.64 μg/gFW (Table 3) and anthocyanins considerably contribute to the DPPH scavenging activity in ripe fruits [28].

The aqueous extracts of peel and pulp of our samples contain 11.53 ± 1.36 μg/gFW and 6.29 ± 0.53 μg/gFW of total anthocyanins (*p* >0.05), values which are lower than the ethanolic extracts, but with higher values of EC_50_ in the DPPH test: 0.59 ± 0.05 mgDW/mL and 0.58 ± 0.03 mgDW/mL respectively (*p* >0.05), for the peel and pulp, approximately 5 times higher than corresponding ethanolic extracts (Table 3).

No statistically significant difference between the pulp and peel values in each of the two types of extracts was observed. Statistically significant difference was found by comparing the aqueous and ethanolic extracts.

### Lipoxygenase test

The same ethanolic extracts of peel and pulp of apple "Pelingo" showed a remarkable inhibition of lipid peroxidation: 0.10 ± 0.01 μgDW/mL and 0.33 ± 0.02 μgDW/mL respectively. The values, expressed as IC_50_ in the 5-lipoxygenase assay are much lower than those of Trolox (11.89 ± 1.22 μgDW/mL) and ascorbic acid (18.63 ± 1.31 μgDW/mL) and almost ten times lower than those of BHT (3.86 ± 0.25 μgDW/mL) (Table 3). This means that the ethanolic extract of red peel and red flesh of our apple are "in vitro" about ten time more active than BHT in counteracting the activity of 5-lipoxygenase with a small difference between the two tested extracts for ethanol extract of peel more rich in total polyphenols and anthocyanins.

By contrast, aqueous extracts of peel and pulp showed values of IC_50_ too high to be considered in the 5-lipoxygenase test: 446 ± 48.7 μgDW/mL and 3688 ± 35.27 μgDW/mL for peel and pulp respectively. These aqueous extracts contain respectively 1.45 mg/gFW and 0.36 mg/gFW of total polyphenols and 11.53 μg/gFW and 6.29 μg/gFW of total anthocyanins (Table 3).

These higher values of IC_50_ in the 5-lipoxygenase test could be due to the strong decrease of the total anthocyanins in the aqueous extracts compared to a smaller decrease of total polyphenols always referred to aqueous extracts, and support the results of Hamauzu et al. [25] which show that the ethanolic apple extract had the strongest activity in the linoleic acid peroxidation system.

In Table 3 statistical analysis for the aqueous extracts were not reported because the results were not significant. Pulp and flesh ethanolic extracts values were statistically different.

### ORAC

Finally (Table 3) the antioxidant capacity of apple extracts when measured by ORAC were 42,97 ± 4.73 and 31.99 ± 3.52 μmol Trolox eq./g FW for aqueous extracts (pulp and peel, respectively). The values obtained for ethanolic extracts were 23.19 ± 2.41 and 44.07 ± 3.74 μmol Trolox eq./g FW (pulp and peel, respectively). For ORAC, antioxidants are evaluated as scavengers of AAPH-derived aqueous peroxyl radicals. Fluorescein loses its fluorescence on oxidation by AAPH, and the presence of antioxidants delay the loss of fluorescence. Several antioxidant assays have been developed over the years and they all use a ROS generator. The ORAC assay is unique in that its ROS generator, AAPH ((2,2^I^-azobis(2-methylpropionamidine) dihydrochloride)), produces a peroxyl free radical upon thermal decomposition that is commonly found in the body, making the reaction biologically relevant. Furthermore, since AAPH is reactive with both water and lipid soluble substances it can be used to measure the total antioxidant potential. The ORAC assay is quickly becoming a standard method by which to measure a substance’s antioxidant capacity. Rupasinghe Vasanta et al. [29] demonstrated that the inhibition of PUFA oxidation was moderately correlated with all of the antioxidant capacity measures thus showing that the antioxidant capacity assays, Folin-Ciocalteau, FRAP and ORAC, could be used in screening fruit extracts prior to their use in food model systems.

In our opinion, particularly interesting is the ORAC value of the aqueous extract of the red pulp of our apple "Pelingo": 42.97 ± 4.73 μmol Trolox eq./g DW, slightly higher than the same value found for the juice of *Prunus spinosa*: 36.0 ± 2.78 and in line with the ORAC values found for small red fruits such as vaccinium (blueberry, huckleberry, cranberry), rubus (raspberries, blackberries) and ribes (currants) [28].

In the determination of ORAC assay the highest values are observed in peel ethanolic extract and in pulp aqueous extract and the results obtained were not significantly different. On the contrary, significant differences were observed between pulp and peel aqueous and ethanolic extracts.

To the best of our knowledge, ORAC data of other red apple pulp have never been reported in literature.

The total polyphenolic content, anthocyanin content and antioxidant activity varied considerably depending on the part of the fruit and on the solvent used for the extraction. Apple peels possessed higher contents of phenolic compounds when compared to flesh with both extraction solvents used. A similar trend in the total phenolic content of apple parts was found among studied cultivars in different countries.

Lamperi et al. [26] studied the polyphenol content (Folin-Ciocalteau method) for peel and flesh respectively of three red apple cultivars from Italy: Annurca, Red Chief and Staynam Neepling; polyphenols correspond to 565 ± 73 and 126 ± 19 for the first cultivar, 576 ± 114 and 104 ± 24 for the second cultivar and 574 ± 78 and 118 ± 9 for the third cultivar; values expressed as mg of (+)-Catechin for 100 g FW (fresh weight).

Likewise Vieira et al. [30] showed that the total phenolic content (mg gallic acid equivalents/100 gr FW) for Fuji apple from Santa Caterina State-Brazil correspond to 577.9 ± 8.99 for peel and 140.9 ± 198 for flesh while in organic Golden Delicious apples from Italy the total polyphenolics (HPLC methods) were 1204 ± 76.2 mg/kg^-1^ FW for peels and 241 ± 30.2 mg/kg^-1^ FW for flesh.

The same authors found that the flesh and the peel of the apples showed a different type and distribution of these phenolics: the flesh contains chlorogenic acid, neochlorogenic acid, caffeic acid, catechins, epicatechins, procyanidins, phloridizin, while the peels, in addition to the above mentioned compounds, have additional phenolics not found in the flesh such as anthocyanins and a high amount of quercetin glycosides.

Tsao et al. [31] reported the total content of anthocyanins in the peel of the following cultivars of red peel and white flesh apples: Empire (208.2 μg/g FW), Cortland (159.8 μg/g FW), Red Delicious (148.9 μg/g FW) and Ida Red (11.0 μg/g FW), while Khanizadeh et al. [32] reported the same results for Mc Intosh Summerland (121.1 μg/g FW), Spartan (286.8 μg/g FW) and Gala (197.7 μg/g FW).

The red peel of Annurca, Red Chief and Stayman Neepling cultivars posses high amounts of anthocyanins: 230.3, 200.2 and 130.7 μg/g FW respectively.

The measured anthocyanin content of the apple peels was related to their appearance. The red color of the apple peels is mainly due to the presence of cyanidin-3-galactoside, the major anthocyanin present in red or partially red genotypes [26,27,32].

## Materials and methods

### Chemicals

All solvents used were HPLC grade; CH_3_CN and MeOH for HPLC were purchased from Merck (Darmstadt, Germany). Formic acid (85% v/v) was provided by Carlo Erba (Milan, Italy). Water was purified by a Milli-Qplus system from Millipore (Milford, MA, USA).

Trolox (6-hydroxy-2,5,7,8-tetramethylchroman-2-carboxylic acid), BHT (butylated hydroxytoluene), ascorbic acid, quercetin, DPPH (1,1-diphenyl-2-picrylhydrazyl radical), 5-lipoxygenase, linoleic acid, AAPH [2,2^I^-azobis(2-amidinopropane) dihydrochloride] and fluorescein sodium salt were purchased from SIGMA (Milano, Italy).

### Standards

For the qualitative analysis the following standards were used: chlorogenic acid, (+)-catechin, (-)-epicatechin, hyperoside, quercetin-3-O-glycoside, quercitrin, rutin and cyanidin-3-O-galactoside. All standards were purchased from Extrasynthèse. For the quantitative analysis the following standards were used: chlorogenic acid, (+)-catechin, quercetin-3-O-glycoside, and cyanidin-3-O-galactoside (purity more than 98%, checked by HPLC and NMR).

### Plant material

The ripe apple fruit samples of apple "Pelingo", (Ministero Delle Politiche Agricole Alimentari e Forestali-Bollettino delle Varietà Vegetali N.3/2011-( http://www.politicheagricole.it/flex/cm/pages/ServeBLOB.php/L/IT/IDPagina/3577) were collected during August 2010 from few scattered plants; found and classified by Prof. Giovanna Giomaro, University of Urbino "Carlo Bo", in Metauro valley (Pesaro-Urbino), Marche region, Italy. The main characteristics of the fruits are: medium size, symmetrical globose shape, medium-long and large petiole, deep and medium wide stalk cavity, smooth skin with few and small lenticels, yellow green ground color, purple red over color covered in bloom, red and medium soft flesh, intense aroma and good taste. The maturity stage of the fruits (2–2.5 on the scale introduced by the Research Centre Laimbourg) was evaluated by the "Potassium Iodine Test" (data not shown) [33]. The fruits were immediately utilized for extraction.

### Extracts preparation

Sixteen ripe fruits constitute the group of samples for extraction; four replications of four apples each were randomly selected and samples consisted of 2 g of peel and 5 g of flesh from the equatorial section of fruit.

The samples were immediately extracted in a mortar using the procedure described by Coseteng and Lee [34] with modification. Briefly, the tissues were extracted twice with a solution of 80% ethanol (ethanol:water 80:20, v/v) or distilled water for 10 and 5 minutes at 80°C and then filtered. Samples were adjusted to 20 mL with 80% ethanol or water and kept at -20°C until use. Furthermore, samples for HPLC analyses were extracted using acidified distilled water or ethanol prepared with 0.5% HCl and centrifuged (1000 X g for 10 min) [34].

### HPLC–DAD analysis instrumentation

The HPLC system consisted of a HP 1100 L instrument with a Diode Array Detector and managed by a HP 9000 workstation (Agilent Technologies, Palo Alto, CA, USA). The column was a Synergy max RP (150 mm × 3.0 mm) with a particle size of 4 μm (Phenomenex) maintained at 27°C. The eluents were H_2_O at pH 2.0 by formic acid 5% (A) and acetonitrile (B) with a flow rate of 0.4 ml/min. The elution method involved a multistep linear solvent gradient changing from an initial concentration of 95% (A) to 78% (A) in 8 min; 5 min to 74% (A); 12 min to 65% (A) then 5 min to initial conditions. The total time of the analysis was 30 min, equilibration time 5 min. Injected volume of the samples was 5 μl solution. The UV–vis spectra were recorded between 220 and 650 nm in both cases. Chromatographic profiles were registered at 254, 280, 330, 350 and 520 nm.

### HPLC–MS analysis instrumentation

The HPLC system described above was interfaced with a HP 1100 MSD API-electrospray (Agilent Technologies, Palo Alto, CA, USA). The same column, time period and flow rate were used during the HPLC–MS analyses. Mass spectrometry operating conditions were optimised in order to achieve maximum sensitivity values: negative and positive ionization mode, scan spectra from *m*/*z* 100 to 1500, was used with a gas temperature of 350°C, nitrogen flow rate of 10 l/min, nebulizer pressure 30 psi, quadrupole temperature 30°C, capillary voltage 3500 V. The applied fragmentors were in the range 60, 120 and 180 V.

### Identification of peaks and peak purity

Identification of all constituents was performed by HPLC–DAD and MS analysis by comparing the retention time, the UV and MS spectra of the peaks in the samples with those of authentic reference samples or isolated compounds and in some cases data reported in the literature. A detailed presentation of the mode of identification is provided in Table 1. The purity of peaks was checked by a Diode Array Detector coupled to the HPLC system, comparing the UV spectra of each peak with those of authentic references samples and/or by examination of the MS spectra.

### Quantitative determination of constituents

The method of external standard was applied to quantify each compound by HPLC-DAD. Quantification of individual constituents was performed using a regression curve, each point in triplicate. Measurements were performed at 280 nm for catechins/procyanidins, at 330 nm for the caffeoylquinic acids, at 350 nm for flavonols and at 520 nm for anthocyanins.

### Total anthocyanin content

The total anthocyanin content of the apple extracts was measured using the differential pH method reported by Elisia et al. [19] and Wrolstad and Giusti [20]. Two aliquots of juice were dissolved separately in potassium chloride buffer (KCL 0.025 M, pH 1.0) and sodium acetate (CH_3_CO_2_Na 3H_2_O, 0.4 M, pH 4.5). The absorbance measurements of the samples were read at 510 and 700 nm against a blank cell containing distilled and deionized water. The absorbance (A) of the diluted sample was then calculated as follows:

$$A\;=\;{\left(A_{510\mathrm{nm}}‒A_{700\mathrm{nm}}\right)}_{\mathrm{pH}1.0}‒{\left(A_{510\mathrm{nm}}‒A_{700}\right)}_{\mathrm{pH}4.5}$$

The monomeric anthocyanin pigment concentration in the original sample was calculated according to the following formula:

$$\mathrm{Anthocyanin}\;\mathrm{content}\;\left(\mathrm{mg}/L\right)=\frac{A\;\times \;\mathrm{MW}\;\times \;\mathrm{DF}\;\times \;1000}{\varepsilon \times \;L}$$

where cyanidin-3-galactoside molecular weight (MW = 449.2) and the absorptivity (ε = 26.900) constans were used [19,20].

### Total polyphenol content

The total content of the polyphenolic compounds was determined by the Prussian Blue method [21] with slight modifications. Aliquots of the juice were made up to 1 mL with distilled water; after adding 60 μL of 0.1 M FeNH_4_(SO_4_)_2_, they were incubated for 20 min at room temperature (22°C). Subsequently, 60 μL of 8 mM K_3_Fe(CN)_6_ were added to the sample, and after 20 min at room temperature the optical density of the mixture was determined at 720 nm (Jasco V-530 spectrophotometer, Tokyo, Japan). Quercetin was used as standard to construct a calibration curve.

### DPPH assay

Radical scavenging activity was determined by a spectrophotometric method based on the reduction of an ethanol solution of 1,1-diphenyl-2-picrylhydrazyl (DPPH), Mellors and Tappel [35].

The DPPH assay was conducted as follows: a 100 μM ethanolic solution of DPPH was prepared and various volumes of sample diluted in distilled water were added to 1.5 mL of this solution. The decreasing absorbance at 517 nm was recorded after 30 min at room temperature (22°C). and the percent decrease (corrected for the control, without addition of antioxidant agents) was taken as an index of the antioxidant capacity. Tests were carried out in triplicate. Trolox (6-hydroxy-2,5,7,8-tetramethylchroman-2-carboxylic acid), BHT (butylated hydroxytoluene) and ascorbic acid were used as positive controls.

The EC_50_ values, defined as the amount of antioxidant necessary to decrease the initial DPPH (radical form) concentration by 50%, were calculated from the results.

### Lipoxygenase test

Inhibition of lipid peroxides formation was evaluated by the 5-lipoxygenase test in the sample and in the positive controls. The activity of the enzyme was assayed spectrophotometrically according to the method of Holman, which was modified by Sud’Ina et al. [36].

The assay mixture (1 mL) contained: 0.1 mM linoleic acid, the sample (or the same quantity of solvent as reference) and 50 mM sodium phosphate, pH 6.8. This mixture was maintained at 23°C for about 5 min.

Subsequently, 0.18 μg mL^-1^ of commercial 5-lipoxygenase was added to the mixture and the formation of hydroperoxides from linoleic acid was observed spectrophotometrically at 235 nm at 23°C.

The IC_50_ values, defined as the amount of antioxidant necessary to inhibit lipid peroxidation by 50%, were calculated from the results.

### Oxygen Radical Absorbance Capacity (ORAC)

The original method of Cao et al. [37] was used with slight modifications. Fluorescein (3^I^,6^I^-dihydroxy-spiro[isobenzofuran-1[3*H*],9^I^[9*H*]-xanthen]-3-one) was chosen as the fluorescent probe instead of B-phycoerythrin (B-PE) [38]. When the sample, with antioxidant activity, had exhausted its capacity to trap peroxyl radicals, that had been induced by 2,2^I^-azobis(2-amidinopropane)dihydrochloride (AAPH) at 37°C, fluorescein became the target of the radicals and lost its fluorescence. The area under the curve (AUC) of fluorescence decay was proportional to the antioxidant capacity of the sample, and a comparative evaluation with Trolox was performed.

The final reaction mixture for the assay (1 mL) was prepared as follows: 825 μL of 0.05 μM fluorescein sodium salt in 0.075 M sodium phosphate buffer, pH 7.0, 100 μL of properly diluted sample in 0.075 M sodium phosphate buffer, pH 7.0 or 50 μM Trolox. The control was 0.075 M sodium phosphate buffer, pH 7.0. The fluorescence was read every 5 min at 37°C using a JASCO FP-6200 spectrofluorometer at 485 nm excitation, 520 nm emission. When stability was reached, the reaction was started with 75 μL of 5.55 mM AAPH and fluorescence was measured every 10 sec until zero fluorescence was detected.

The ORAC value is calculated according to the formula:

ORAC (μmol Trolox equivalent/g DW) = [(A_S_ - A_B_)/(A_T_ - A_B_)] *kah,* where A_S_ is the AUC of fluorescein in the presence of the sample calculated with Spectra Manager for Windows 95/NT (Spectra Analysis) program, A_T_ is the AUC of the Trolox, A_B_ is the AUC of the control, *k* is the dilution factor, *a* is the concentration of Trolox in mmol/L and *h* is the ratio between the litres of extract and the grams of sample used for the extraction.

### Statistical analysis

All data are the average of triplicate analyses. Calculation were performed with the GraphPad Prism program [GraphPad Software, Inc., USA]. One–way analysis of variance was performed followed by the Bonferroni’s multiple comparison test; *p* values of <0.05 were regarded as significant and *p* values of <0.01 very significant.

## Conclusion

In conclusion the results showed that our apple contained an appreciable amount of polyphenols also in the flesh. The red color of pulp and the fruity-sweet flavor distinguish it from other types of wild apples with red flesh that often result not tasty or sour and therefore not marketable. The spontaneous production of red pigments in the pulp of "Pelingo" apple is a very interesting prospect in the international market and food consumption since apples are generally consumed without the peel, loosing the healthy anthocyanins and this new variety can also be useful for researchers engaged in apples varietal innovation.

## Abbreviations

HPLC-DAD-MS: High-performance liquid chromatography-diode array detector-mass spectrometry.

## Competing interests

The authors declare that they have no competing interests.

## Authors’ contributions

GG Has discovered and patented the apple studied and made a significant contribution to drafting and revising the manuscript. AK^+^ Made a substantial contribution to acquisition of data, analysis, drafting of the manuscript. ARB Made a significant contribution to drafting and revising the manuscript. AB^+^ Made a substantial contribution to analysis, acquisition of data, drafting of the manuscript. LG Made a significant contribution to interpretation of data, to drafting and revising the manuscript. DR Made a significant contribution to drafting and revising the manuscript. DF Has made a substantial contribution to conception and design, drafting and revising the manuscript for intellectual content. All authors read and approved the final manuscript.
